# Effect of Ball-Milling Process on Microwave Absorption Behaviors of Flaky Carbonyl Iron Powders

**DOI:** 10.3390/ma16124397

**Published:** 2023-06-15

**Authors:** Siyuan Yang, Fei Wang, Zhe Zhang, Zhiming Liu, Jiliang Zhang, Kaiyong Jiang

**Affiliations:** 1 Fujian Key Laboratory of Special Energy Manufacturing, Huaqiao University, Xiamen 361021, China; 1611121038@stu.hqu.edu.cn (S.Y.); zhangzhehqu@foxmail.com (Z.Z.); mikeswell@hqu.edu.cn (J.Z.); jiangky@hqu.edu.cn (K.J.); 2 Xiamen Key Laboratory of Digital Vision Measurement, Huaqiao University, Xiamen 361021, China; 3 School of Information Engineering, Nanchang University, Nanchang 330031, China; zhimingliu@ncu.edu.cn

**Keywords:** carbonyl iron powders, absorption performance, ball-milling time, ball-milling rotation speed, electromagnetic parameters

## Abstract

Electromagnetic (EM) wave absorption performance is greatly affected by the microscopic morphology of the absorbing material particles. In this study, a facile and efficient ball-milling method was applied to increase the aspect ratio of particles and prepare flaky carbonyl iron powders (F-CIPs), one of the most readily commercially available absorbing materials. The effect of ball-milling time and rotation speed on the absorption behaviors of the F-CIPs was investigated. The microstructures and compositions of the F-CIPs were determined using scanning electron microscopy (SEM) and X-ray diffraction (XRD). The EM parameters were measured using a vector network analyzer (VNA) in the frequency range of 2–18 GHz. The results indicated that the ball-milled flaky CIPs exhibited a better absorption ability than the raw spherical CIPs. Among all the samples, the sample milled at 200 r/min for 12 h and the sample milled at 300 r/min for 8 h showed remarkable EM parameters. The ball-milling sample with 50 wt.% F-CIPs had a minimum reflection loss peak of −14.04 dB at a thickness of 2 mm and a maximum bandwidth (RL < −7 dB) of 8.43 GHz at a thickness of 2.5 mm, a result that conformed with the transmission line theory. Hence, the ball-milled flaky CIPs were considered to be beneficial for microwave absorption.

## 1. Introduction

With the development of communication technology, electromagnetic (EM) waves permeate all aspects of human activities. Although convenient in our daily lives, this brings EM radiation, EM interference, and other increasingly prominent adverse effects [1,2]. Absorbing materials, which can effectively prevent these adverse effects [3,4], have gradually become a focus of research, and they have been widely used in the construction [5,6], military [7,8], and communication fields [9], among others. Therefore, it would be of great significance to develop a coating with a stronger absorption ability [10]. The EM-absorbing performance of a coating mainly depends on the EM-absorbing agent. According to different EM loss mechanisms, EM-absorbing agents can be divided into three types: dielectric loss, resistance loss, and magnetic loss. Magnetic loss-absorbing materials, such as carbonyl iron powder [11], ferrite [12,13,14], and other metal micro powders [15], may be considered for their impedance matching and absorbing ability, and these materials have been widely used in single-type absorbing coatings. Among them, carbonyl iron powders (CIPs), which have the advantages of high saturation magnetization, high permeability, and high Curie temperatures [16,17], are one of the most commonly used types of EM-absorbing agents.

The EM characteristics of CIPs are greatly affected by their microscopic morphology [18,19,20], an aspect that has received extensive attention from researchers. Yang et al. [21] found that, compared with spherical CIPs, the maximum reflection loss of dendritic CIPs increased by 94% (i.e., up to −47.14 dB), and their research showed that an anisotropic structure is an effective way to improve the dielectric constant and dielectric loss of materials. Through annealing and selective point etching, Wang et al. [22] prepared porous chip CIPs which had a large specific surface area and high-frequency permeability. Walser [23] found that a high aspect ratio and anisotropic shape enabled flaky materials to overcome the Snoek limit and improved their resonance frequency and high-frequency permeability, thus obtaining excellent EM-absorbing performances.

At present, the methods for preparing flaky metal particles include the reduction method and mechanical ball milling [24,25]. Mechanical ball milling is widely applied because it requires simple equipment and enables easy parameter control [26,27]. In this method, the powder particles undergo deformation, fracture, and welding processes repeatedly through the collisions that occur between the tank, the ball, and the powder, and a flaky sample is ultimately produced. Ball-milling time and rotation speed are the two most important factors affecting the final quality of the samples produced via this process [28]. Therefore, it is necessary to investigate the effects of these two factors in order to obtain a process suitable for the mass production of flaky particles with excellent EM-absorbing performance.

In this work, the influence of ball-milling time and rotation speed on the morphology, composition, and EM parameters of CIPs was studied. The effects of these ball-milling parameters on the EM-absorbing properties of composite coatings with different thicknesses were analyzed in accordance with the transmission line theory, and an optimal CIP ball-milling process for the preparation of broadband high-efficiency EM-absorbing coatings was developed.

## 2. Experimental

### 2.1. Materials

**Figure 1:**
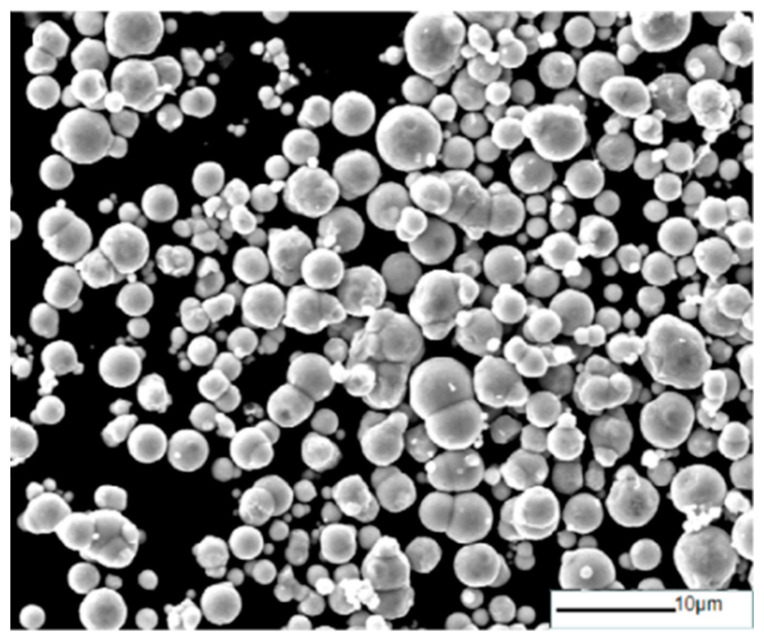
SEM image of raw CIP.

Raw CIP (shown in Figure 1), consisting of particles that exhibited regular spherical shapes with particle diameters of about 3–5 μm, was provided by Lebo Metal Material Technology Co., Ltd. Shijiazhuang, China. Anhydrous ethanol (EtOH, AR) and N-hexane (AR) were provided by Sinopharm Chemical Reagent Co., Ltd. Shanghai, China. Paraffin sections (pathological grade, melting point 58–60 °C) were provided by Aladdin Biochemical Technology Co., Ltd., Shanghai, China.

### 2.2. Preparation of Flaky CIP

At a ball-to-material ratio of 10:1, zirconia grinding balls (with diameters of 5 mm, 8 mm, and 10 mm added at a ratio of 2:3:5, respectively) and the spherical carbonyl iron powder were placed in a 100 mL zirconia ball-milling tank, and anhydrous ethanol was added to fill one-third of the ball-milling tank. The ball mill tank was then placed in a planetary ball mill (YXQM-4L, Changsha Miqi Instrument Equipment Co., Ltd., Changsha, China). After ball milling, the powder was removed, washed with anhydrous ethanol, and dried in a vacuum oven at 50 °C for 4 h. Finally, the flaky CIP was collected and tested.

### 2.3. Selection of Ball-Milling Parameters

According to the results of the previous experiment, the parameters listed in Table 1 and Table 2 were selected to study the influence of ball-milling time and rotation speed on microwave absorption behaviors of flaky CIPs.

### 2.4. Test and Characterizations

The morphology of the powders was observed by scanning electron microscopy (SEM, JEOL, JSM-IT500LA, Tokyo, Japan). The phase composition was analyzed by Cu Kα radiation X-ray diffraction (XRD, SmartLaSmar, Rigaku, Tokyo, Japan) with a scanning speed of 10°/min, a voltage of 40 kV, a current of 20 mA, and a scanning range of 20–90°. The complex permittivity (real part ε′ and imaginary part ε″) and complex permeability (real part μ′ and imaginary part μ″) were measured using the coaxial transmission line method and a vector network analyzer (VNA, E5071C, Keysight Agilent, Santa Clara, CA, USA) at 2–18 GHz. The preparation process of the sample for the transmission line coaxial method is as follows: First, paraffin sections and CIPs were added to a grinding bowl in a mass ratio of 1:1 (50 wt.% F-CIPs). Then, a small amount of N-hexane was added to dissolve the paraffin and the mixture was thoroughly ground until they were completely mixed. Afterward, the mixture was left sitting for one day until the n-hexane completely evaporated. Finally, a pressing machine and a coaxial circular mold were used to prepare a consolidated ring sample with an outer diameter of 7.0 mm, inner diameter of 3.0 mm, and height of approximately 2 mm.

## 3. Results and Discussion

### 3.1. Effect of Ball-Milling Parameters on Microstructure of F-CIPs

**Figure 2:**
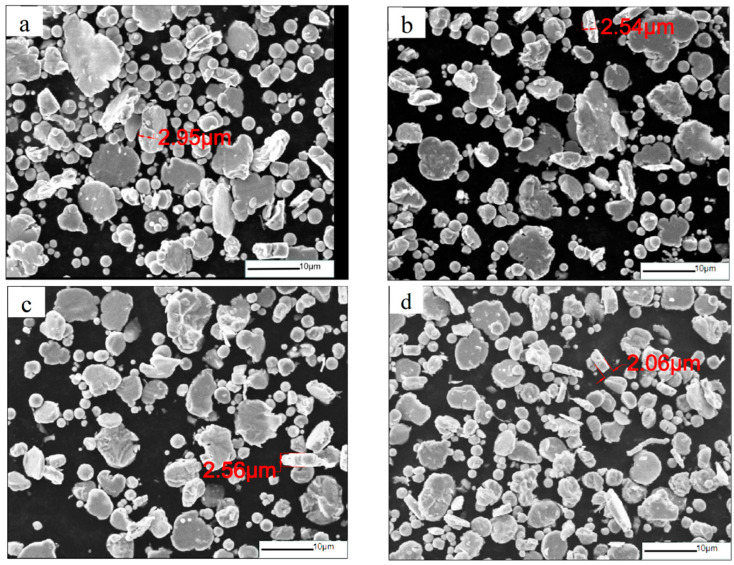
SEM images of CIPs milled for (**a**) 4 h; (**b**) 8 h; (**c**) 12 h; (**d**) 16 h.

Figure 2 shows the morphology of CIPs milled at 200 r/min for different ball-milling times. The CIPs milled for 4 h and 8 h had already started to flatten, but a large number of particles still maintained a spherical shape, and the distribution of particle size was obviously uneven, as shown in Figure 2a,b. Figure 2c,d, respectively, show the microstructure of the CIPs milled for 12 h and 16 h. The number of flattened particles increased significantly with prolonged milling time, but the 16 h sample contained many small particles, which may indicate an excessive ball-milling time. In general, the thickness of the flaky particles in the 12 h and 16 h samples was already lower than the skin depth of the CIPs at low-frequency bands, which can effectively suppress the eddy current loss of the material, reduce the reflection of EM waves on its surface, and indirectly improve the absorbing performance of the material.

**Figure 3:**
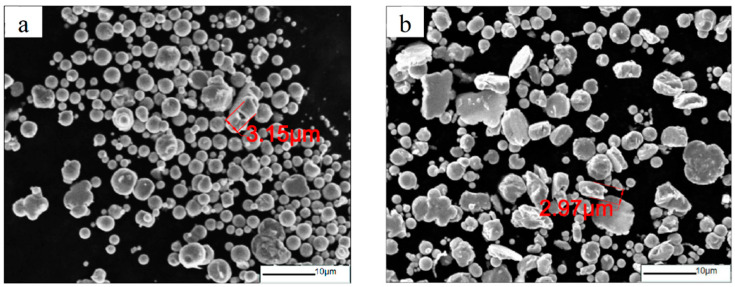

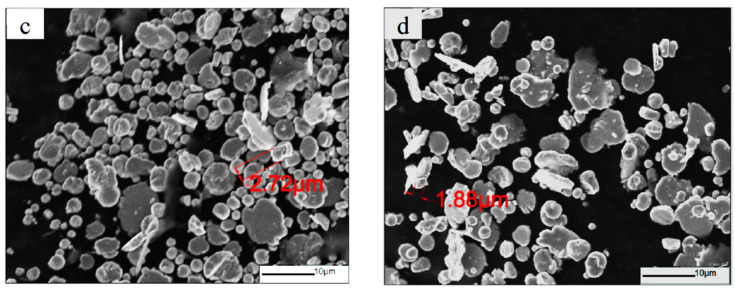
SEM images of CIPs ball-milled at (**a**) 150 r/min; (**b**) 200 r/min; (**c**) 250 r/min; (**d**) 300 r/min.

Figure 3 shows the morphology of CIPs at different ball-milling rotation speeds for 8 h. Figure 3a shows that the most of CIPs milled at 150 r/min still remained spherical. This indicates that the milling energy was too low to cause deformation of the particles. From Figure 3b,c, it can be seen that with the increase in rotation speed, there were significant changes in the microstructure of the carbonyl iron powder, and most of the particles underwent a transformation to a flaky shape, while the thickness of the flakes also decreased significantly. When the rotation speed increased to 300 r/min, the CIP particles showed an obviously flaky structure, and the anisotropy of the CIP shape significantly increased, as shown in Figure 3d. Overall, the increase in rotation speed promoted the flakiness of the CIP particles. Meanwhile, there was no obvious particle breakage.

### 3.2. Effect of Ball-Milling Parameters on Phase Composition of F-CIPs

**Figure 4:**
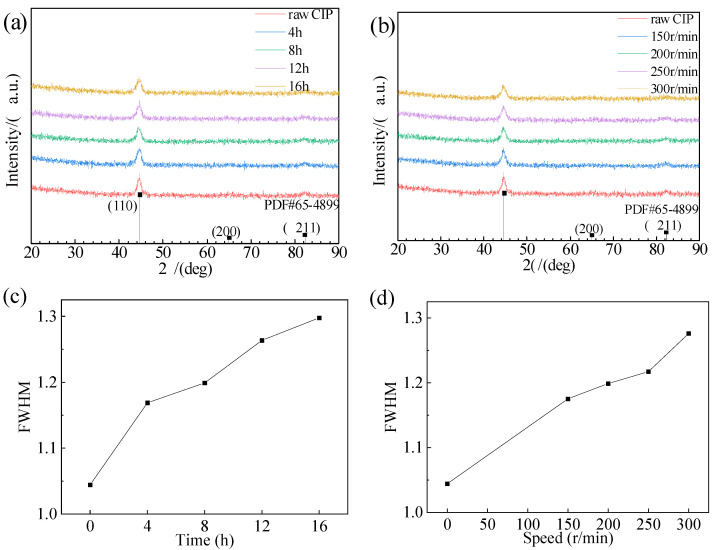
X-ray diffraction patterns of CIPs (**a**) for different ball-milling times; (**b**) at different ball-milling rotation speeds; FWHM of CIPs: (**c**) for different ball-milling times; (**d**) at different ball-milling rotation speeds.

The X-ray diffraction patterns of raw CIPs and ball-milled CIPs are displayed in Figure 4. All the samples had three diffraction peaks at 2θ = 44.67°, 65.02°, and 82.33° corresponding to the (110), (200), and (211) planes of α-Fe. Figure 4c,d show the full width at half-maximum (FWHM) of the XRD patterns of different samples of CIPs. From Figure 4c,d, it can be observed that the FWHM of the sample increased with increasing ball-milling time or speed, indicating that the grain size of the CIPs decreased. However, the identical diffraction peaks of the samples indicate that, as the ball-milling time or rotation speed increases, there was no change in the crystal phase or composition of the CIPs.

### 3.3. Effect of Ball-Milling Parameters on EM Properties of F-CIPs

**Figure 5:**
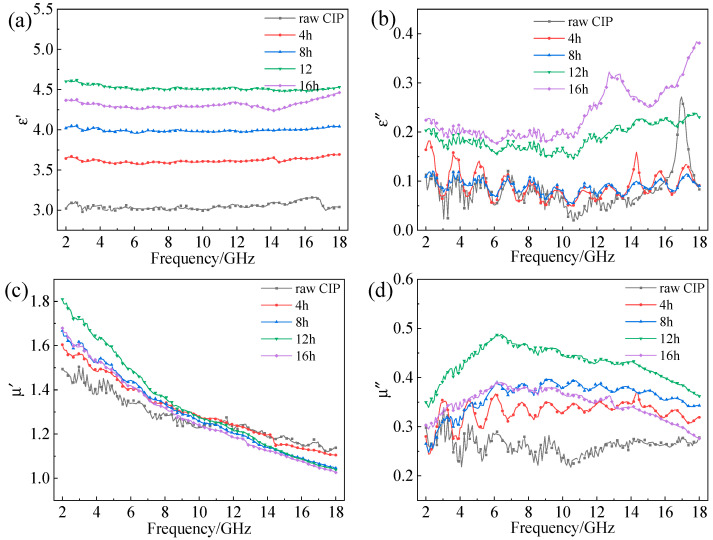
EM parameters versus frequency of CIPs at different ball-milling times: (**a**) ε′; (**b**) ε″; (**c**) μ′; (**d**) μ″.

The frequency dispersion of the complex permittivity and permeability of CIPs after different ball-milling times are shown in Figure 5. From Figure 5a, it can be seen that the ε′ of the CIP/paraffin sample remained essentially unchanged with changes in frequency, but with the increase in ball-milling time, the ε′ initially increased and then slightly decreased. This is due to the variation in the CIP aspect ratio which greatly increases the interfacial polarization of the material, thus improving the ε′. However, a prolonged milling time will lead to the breakage of particles, as observed in Figure 2d, thereby reducing the ε′. As can be seen from Figure 5b, the ε″ of the CIPs ball-milled for 0–8 h has no significant distinction. However, the ε″ of CIPs ball-milled for 12 h and 16 h obviously improved. This can be explained by the free electron theory [18,29]:

(1) $$\varepsilon \prime \prime =\sigma /2\pi \varepsilon_{0}f$$

where σ and $\varepsilon_{0}$ are the conductivity and permittivity in free space, respectively. At a certain electromagnetic frequency f, the ε″ is proportional to the conductivity σ of the material. According to the SEM images in Figure 3, the samples milled for 12 h and 16 h had higher aspect ratios; these are more likely to form a conductive network inside the material, resulting in a higher conductivity of the material, thus increasing the ε″ of the CIPs.

μ′ showed a good agreement with the frequency dispersion, and its change was not obvious compared to the μ″ permittivity (see Figure 5c,d). The largest μ′ value for all samples was 2 after milling for 12 h. The μ′ values decreased dramatically in the frequency range of 1–10 GHz, which may be due to domain-wall resonance and relaxation. μ′ increased with increasing ball-milling time at a low-frequency band, but the difference was very small. On the contrary, at a high-frequency band, the μ′ decreased with increasing ball-milling time.

With the extension of milling time, the μ″ first increased and then decreased slightly; the maximum μ″ value was achieved after milling for 12 h. μ″ is related to the magnetic crystal anisotropy and morphological anisotropy of the particles. The increased flattening degree enables the CIPs to break through the Snoek limit [30,31], which increases the peak of the low-frequency permeability of the material (corresponding to the natural resonance peak of the material) on the one hand, and slows down the decrease in the high-frequency permeability of the material on the other hand. It is worth noting that both μ′ and μ″ of the 16 h ball-milled sample are inferior to that of the 12 h ball-milled sample. It is proposed that an extensive ball-milling time causes over-crushing of the particles which reduces the aspect ratio.

**Figure 6:**
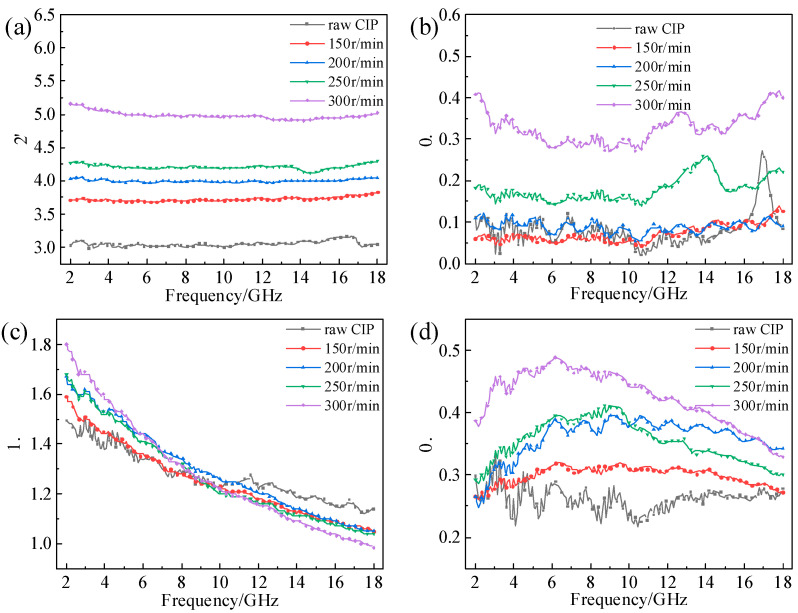
EM parameters versus frequency of CIPs at different ball-milling rotation speeds: (**a**) ε′; (**b**) ε″; (**c**) μ′; (**d**) μ″.

The complex permittivity and permeability spectra of the CIPs under different ball-milling rotation speeds are shown in Figure 6. The variation law of the EM parameters in Figure 6 is similar to that in Figure 5. The ε′ and the ε″ increased with increasing ball-milling rotation speed. All this was due to the improvement in the aspect ratios. In the range of 2–9 GHz, μ′ increased with the increase in milling rotation speed, and in the range of 9–18 GHz, μ′ decreased with the increase in milling rotation speed. The μ″ increased with the increase in milling rotation speed.

**Figure 7:**
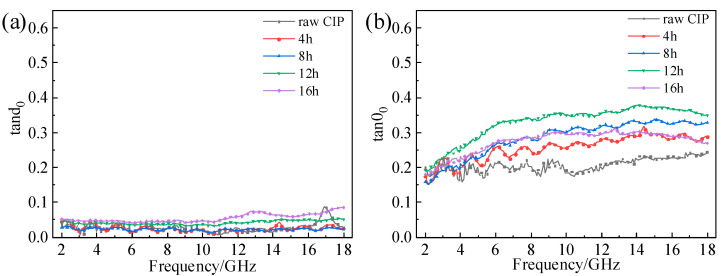
Loss angle tangent versus frequency of CIPs at different ball-milling times: (**a**) $\tan\delta_{\varepsilon }$; (**b**) $\tan\delta_{\mu }$.

**Figure 8:**
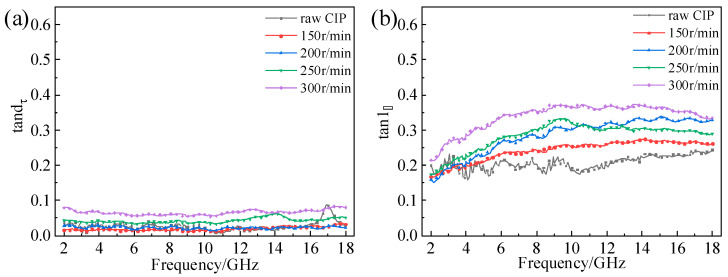
Loss angle tangent versus frequency of CIPs at different ball-milling rotation speeds: (**a**) $\tan\delta_{\varepsilon }$; (**b**) $\tan\delta_{\mu }$.

In general, the tangent of the dielectric loss angle ($\tan\delta_{\varepsilon }=\varepsilon \prime \prime /\varepsilon \prime$) is used to indicate the electrical loss capacity, and the tangent of the magnetic loss angle ($\tan\delta_{\mu }=\mu \prime \prime /\mu \prime$0) is used to indicate the magnetic loss capacity [32,33]. Figure 7 and Figure 8, respectively, show the CIPs’ loss angle tangent versus frequency at different ball-milling times and rotation speeds. It can be seen from Figure 7a that $\tan\delta_{\varepsilon }$ increased with the increase in milling time. In addition, the $\tan\delta_{\varepsilon }$ values of the different samples were basically unchanged between 2 and 18 GHz. $\tan\delta_{\mu }$ first increased and then decreased with the increase in ball-milling time, and the maximum value was reached at 12 h, as shown in Figure 7b. Otherwise, the tangent of the magnetic loss angle first increased and then decreased between 2 and 18 GHz. Comparing Figure 7a with Figure 7b, it can be found the $\tan\delta_{\mu }$ values of all samples were much larger than those of $\tan\delta_{\varepsilon }$. This shows that the absorption of microwaves at 2–18 GHz is dominated by magnetic loss. The results in Figure 8 are similar to those in Figure 5, so they will not be described in detail.

**Figure 9:**
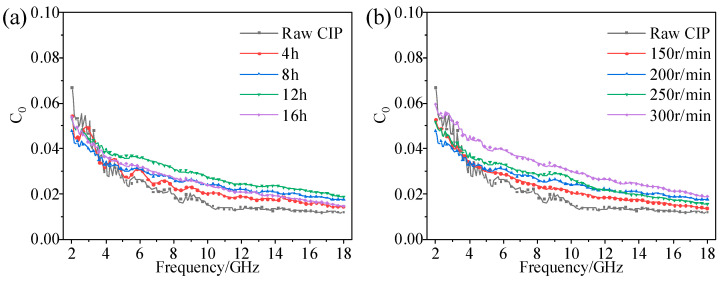
Eddy current loss factor $C_{0}$ versus frequency of CIPs with (**a**) different ball-milling times; (**b**) different ball-milling rotation speeds.

Generally, magnetic loss mainly comes from hysteresis loss, domain wall resonance, natural resonance, exchange resonance, and eddy current loss. The domain wall resonances mainly occur in the MHz range. As a soft magnetic material, CIPs have a trifling hysteresis loss, so the contribution of the two factors to the magnetic loss is negligible. The exchange resonances mainly occur above 10 GHz. The formula for the eddy current loss is as follows [34]:

(2) $$C_{0}=\mu \prime \prime {\left(\mu \prime \right)}^{-2}f^{-1}=\frac{2\pi \mu_{0}\sigma d^{2}}{3}$$

where $C_{0}$ is the eddy current loss factor; μ′ and μ″ are the real and imaginary parts of permeability; and f, $\mu_{0}$, σ, and d are the frequency, vacuum permeability, dielectric constant, and particle thickness, respectively. According to the formula, if the magnetic loss is mainly caused by eddy current loss, $C_{0}$ should be a constant that does not change with frequency. Figure 9a,b show the frequency variation curves of the eddy current loss coefficient of CIPs with different ball-milling times and rotation speeds. As we can see, the evolution law of the two graphs is almost the same. The eddy current loss coefficient declined moderately at a frequency of 2–12 GHz, and decreased slightly or remained constant at 12–18 GHz. This shows that the magnetic loss at 2–12 GHz includes natural resonance and eddy current loss, and the eddy current loss is the main magnetic loss at 12–18 GHz. Moreover, the eddy current loss coefficient at the low-frequency band decreased after ball milling, indicating it is reduced by ball milling, which was consistent with the Snoek limit and skin depth theories.

### 3.4. Effect of Ball-Milling Parameters on Absorbing Behaviors of F-CIPs

In general, the absorbing properties of composites can be described by reflection loss (RL). Based on the transmission line theory, the calculation formula for RL is as follows [35,36]:

(3) $$RL=20\mathrm{lg}\left|\frac{Z_{ink}-1}{Z_{ink}+1}\right|$$

(4) $$Z_{in}=Z_{0}\sqrt{\frac{\mu_{r}}{\varepsilon_{r}}}\tanh\left[j\left(\frac{2\pi fd}{c}\right)\sqrt{\mu_{r}\varepsilon_{r}}\right]$$

where c is the velocity of light, f is frequency, d is the absorber thickness, $\varepsilon_{r}$ is the relative complex permittivity, and $\mu_{r}$ is the relative complex permeability. Generally, the absorption peak value and the effective absorption bandwidth (EAB) are the two indicators of EM-absorbing performance. Here, the EAB was defined as RL < −7 dB, which means the absorption of EM waves exceeds 80%.

**Figure 10:**
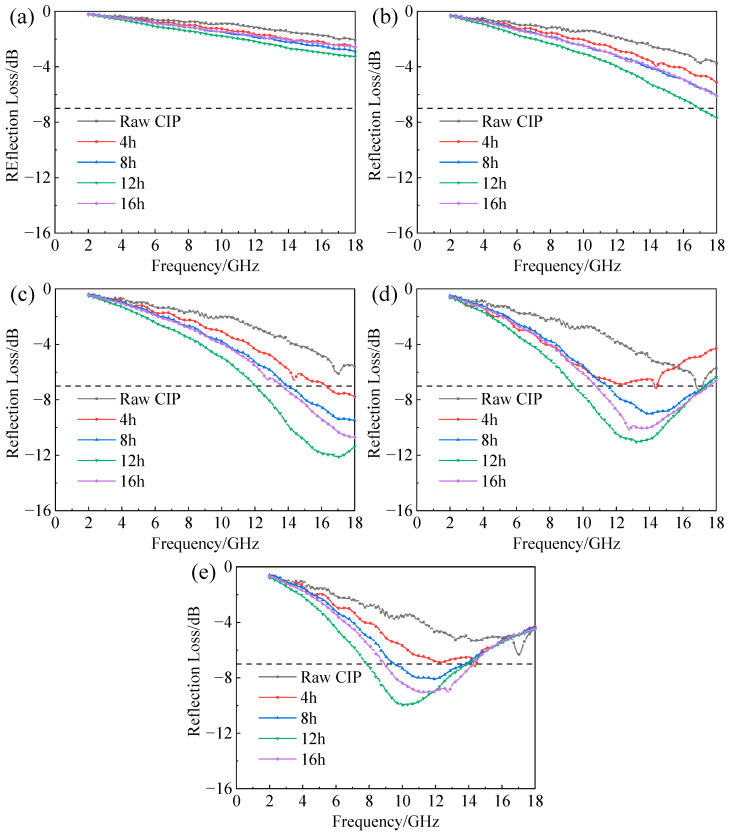
Reflection loss versus frequency of CIPs ball-milled for different times with thicknesses of (**a**) 1 mm; (**b**) 1.5 mm; (**c**) 2.0 mm; (**d**) 2.5 mm; (**e**) 3.0 mm.

Figure 10 shows the influence of ball-milling time on the reflection loss of CIPs/paraffin samples with different thicknesses.

It can be seen that the absorption peaks of all samples shifted to a lower frequency with the increase in thickness. This phenomenon could be explained by the quarter-wavelength theory. The relationship between thickness and absorption peak frequency is as follows [37]:

(5) $$d=\frac{n\lambda }{4}=nc/(4f\sqrt{\varepsilon_{r}\mu_{r}})$$

where d is the absorber thickness, λ is the wavelength, and n=1,3,5…… Combined with the SEM images, it was found that the absorption performance of CIPs was closely related to their aspect ratios and EM parameters. The 12 h sample in Figure 10d had the best absorbing properties at different thicknesses. It can be seen from Figure 10a,b that the wave absorption performance of each sample was poor with almost no EAB when the thickness was 1 mm or 1.5 mm. When the thickness increased to 2 mm, the EAB and absorption peak values of each material were significantly improved. Among them, the ball-milled 12 h sample was very prominent, since RL < −7 dB from 12 GHz to 18 GHz, and the absorption peak value reached −12.11 dB. As the thickness increased to 2.5 mm (Figure 10d), the EAB of the samples in each group continued to increase, and the maximum EAB of the 12 h sample was 9.48–17.36 GHz, but the absorption peak value decreased slightly to −11.03 dB. When the thickness was 3 mm (Figure 10e), the EAB and absorption peak of each sample decreased significantly. The EAB and absorption peak value of the sample first increased and then decreased with the increasing thickness from 1 mm to 3 mm.

In addition, it can be noticed that the EAB of the raw CIP was almost 0 for all thicknesses. On the contrary, the EAB of the samples milled for 8 h, 12 h, and 16 h reached more than 4 GHz if the thickness was greater than 1.5 mm. The samples ball-milled for 12 h had better reflection loss at all thicknesses. When the thickness was 2 mm, the absorption peak was −12.11 dB, which is more than 100% higher than that of the sample of raw CIP. In summary, the results indicated that the absorption ability of ball-milled flaky powders significantly exceeded those of the pristine spherical powders.

**Figure 11:**
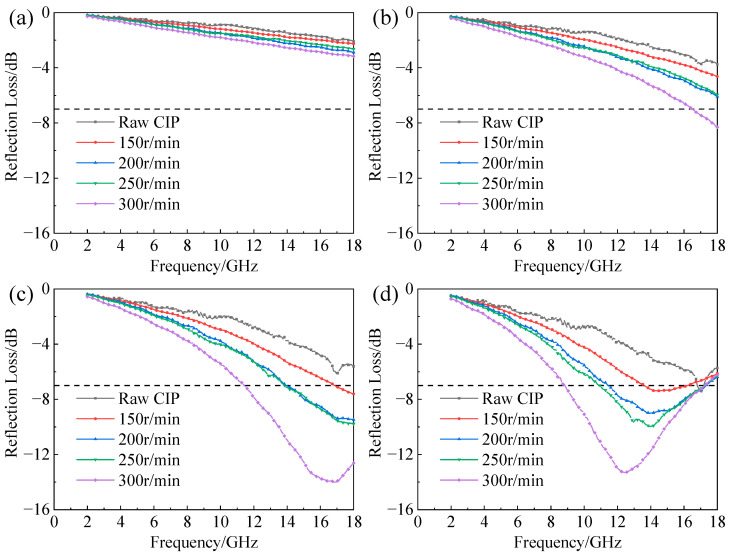

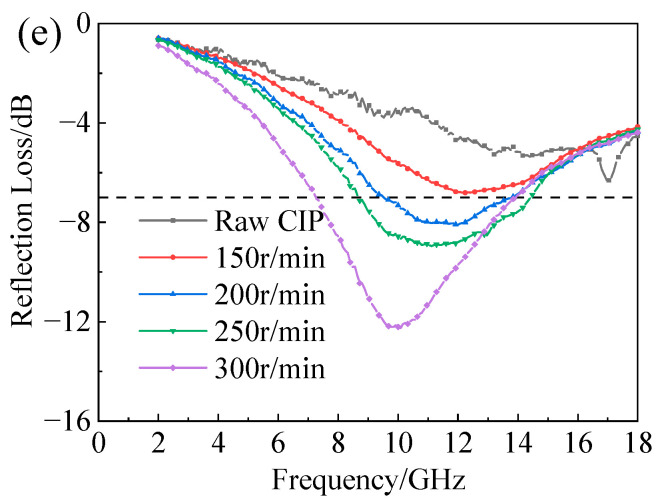
Reflection loss versus frequency of CIPs ball-milled at different rotation speeds with thicknesses of (**a**) 1 mm; (**b**) 1.5 mm; (**c**) 2.0 mm; (**d**) 2.5 mm; (**e**) 3.0 mm.

Figure 11 shows the influence of ball-milling rotation speed on the reflection loss of CIPs/paraffin samples at different thicknesses. The evolution law of RL in Figure 11 is similar to that in Figure 10. With the thickness tuning from 1 mm to 3 mm, the EAB and absorption peak value of all samples showed a trend of first increasing and then decreasing. The RL of each sample was poor at 1 mm and 1.5 mm. The sample ball-milled at a rotation speed of 300 r/min had the best wave absorption performance. When the thickness was 2 mm, the EAB reached 6.3 GHz and the minimum absorption value was −14.04 dB. Then the thickness was 2.5 mm, and the EAB increased to 8.43 GHz with an absorption peak value of −13.3 dB.

**Figure 12:**
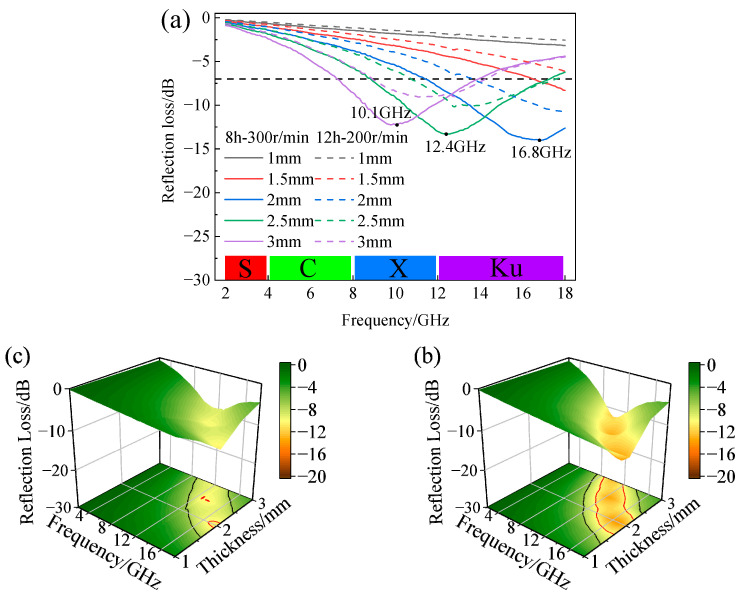
Comparison of the absorption performance of the best two groups: (**a**) reflection loss diagram comparison of f-CIPs with different thicknesses; (**b**) three-dimensional diagrams of reflection loss of F-CIPs milled for 8 h at 300 r/min; (**c**) three-dimensional diagrams of reflection loss of F-CIPs milled for 12 h at 200 r/min.

Based on the above analysis, the CIPs obtained after milling for 12 h at 200 r/min and 8 h at 300 r/min had the best wave absorption performances among the two groups, respectively. To identify the optimal ball-milling parameters for future research, Figure 12 presents a comparison of the RL at thicknesses of 1–3 mm between the two sets of ball-milling conditions. In Figure 12a, for any thickness, the RL of F-CIPs ball-milled for 8 h at 300 r/min was stronger than that of F-CIPs ball-milled for 12 h at 200 r/min. Combined with the color scale, it can be observed that the absorption by F-CIPs was mainly effective for the X band and Ku band. The frequency at which the absorption peak is given in the figure. Three-dimensional diagrams of the RL of the two samples with different thicknesses are shown in Figure 12b,c. On the bottom projection surface of the figure, the region of RL < −7 dB is inside the black curve, and the part of RL < −10 dB (microwave absorptivity exceeding 90%) is inside the red curve. The results demonstrate again that F-CIPs ball-milled for 8 h at 300 r/min had a wider EAB and stronger absorption performance.

Table 3 shows the absorbing performance comparison between the flaky CIPs in produced in this study and previous studies. The EAB performance of CIP tablets was better, but its absorption peak was average. Subsequently, it can be used with other materials as two-component absorbing materials to meet the requirements of having a strong absorption peak.

## 4. Conclusions

The method of ball milling was used to prepare flaky carbonyl iron powders, and the effects of ball-milling time and rotation speed on the absorbing performance were studied. The results show that the aspect ratios increased gradually with increasing ball-milling time and rotation speed, and significantly affect the complex permittivity and complex permeability of the F-CIPs produced. However, the flattened degree of the particles was the result of the coupling effect of two factors; therefore, an excessive ball-milling time and rotation speed will lead to fracture and fragmentation.

According to the calculation of material reflection loss based on the transmission line theory, the effective absorption bandwidth and minimum absorption value were compared using different ball-milling parameters and coating thickness. Consequently, ball-milled CIPs exhibited considerably enhanced microwave absorbing abilities when compared with those of pristine CIPs.

Meanwhile, the F-CIPs ball-milled for 12 h at 200 r/min and 8 h at 300 r/min showed satisfactory microwave absorption abilities. For the sample milled for 12 h at 200 r/min, when the coating thickness was 2 mm, the minimum absorption value reached −12.12 dB, and the EAB (RL< −7 dB) was 12–18 GHz. For the sample milled for 8 h at 300 r/min, when the coating thickness was 2 mm, the minimum absorption value reached −14.04 dB, and the EAB covered 11.7–18 GHz. When the thickness was increased to 2.5 mm, the EAB reached 8.43 GHz.

In general, ball milling to produce flaky particles is a facile and effective means to improve the absorbing performance of CIPs. The absorbing frequency band, bandwidth, and peak value of the F-CIPs can be accurately controlled by adjusting the ball-milling time and rotation speed, which has important research value and application prospects.

## Figures and Tables

**Table 1:** Experimental design for optimizing ball-milling time.

Time	4 h	8 h	12 h	16 h
rotation speed	200 r/min	200 r/min	200 r/min	200 r/min

**Table 2:** Experimental design for optimizing ball-milling rotation speed.

Time	8 h	8 h	8 h	8 h
rotation speed	150 r/min	200 r/min	250 r/min	300 r/min

**Table 3:** Absorbing performance comparison with other CIP-based composites.

Absorber and Content	EAB/GHz (RL < −10 dB)	Minimum RL/dB	Thickness at Minimum RL/mm	Reference
Dendritic CIP (60%)	5.2	−47.14	2.68	[19]
Porous carbonyl iron flakes (20%)	2	−28	2	[20]
Hollow carbonyl iron (55.5%)	3	−11	1	[38]
Flaky CIPs (50%)	4.5	−14	2	This work

## Data Availability

Not applicable.
